# 8-Alkylcoumarins from the Fruits of *Cnidium monnieri* Protect against Hydrogen Peroxide Induced Oxidative Stress Damage

**DOI:** 10.3390/ijms15034608

**Published:** 2014-03-17

**Authors:** Chi-I Chang, Wan-Chiao Hu, Che-Piao Shen, Ban-Dar Hsu, Wei-Yong Lin, Ping-Jyun Sung, Wei-Hsien Wang, Jin-Bin Wu, Yueh-Hsiung Kuo

**Affiliations:** 1 Department of Biological Science and Technology, National Pingtung University of Science and Technology, Pingtung 912, Taiwan; E-Mail: changchii@mail.npust.edu.tw; 2 Department of Chemistry, National Taiwan University, Taipei 106, Taiwan; E-Mail: r93223083@ntu.edu.tw; 3 Institute of Bioinformatics and Structural Biology, National Tsing Hua University, Hsinchu 300, Taiwan; E-Mails: d918214@oz.nthu.edu.tw (C.-P.S.); bdhsu@life.nthu.edu.tw (B.-D.H.); 4 Department of Medical Research, China Medical University Hospital, Taichung 404, Taiwan; 5 Graduate Institute of Integrated Medicine, China Medical University, Taichung 404, Taiwan; 6 Graduate Institute of Marine Biotechnology and Department of Life Science and Institute of Biotechnology, National Dong Hwa University, Pingtung 944, Taiwan; E-Mail: pjsung@nmmba.gov.tw; 7 National Museum of Marine Biology and Aquarium, Pingtung 944, Taiwan; E-Mail: whw@mail.nsysu.edu.tw; 8 Department of Marine Biotechnology and Resources, National Sun Yat-sen University, Kaohsiung 804, Taiwan; 9 Graduate Institute of Pharmaceutical Chemistry, China Medical University, Taichung 404, Taiwan; E-Mail: jbwu@mail.cmu.edu.tw; 10 Department of Chinese Pharmaceutical Sciences and Chinese Medicine Resources, China Medical University, Taichung 404, Taiwan; 11 Department of Biotechnology, Asia University, Taichung 413, Taiwan

**Keywords:** Chinese herb, *Cnidium monnieri*, 8-alkylcoumarin, oxidative stress

## Abstract

Three new 8-alkylcoumarins, 7-*O*-methylphellodenol-B (**1**), 7-methoxy-8-(3-methyl-2,3-epoxy-1-oxobutyl)chromen-2-one (**2**), and 3′-*O*-methylvaginol (**3**), together with seven known compounds (**4**–**10**) were isolated from the fruits of *Cnidium monnieri.* Their structures were determined by detailed analysis of spectroscopic data and comparison with the data of known analogues. All the isolates were evaluated the cytoprotective activity by MTS cell proliferation assay and the results showed that all the three new 8-alkylcoumarins exhibited cytoprotective effect on Neuro-2a neuroblastoma cells injured by hydrogen peroxide.

## 1. Introduction

**Figure 1.**
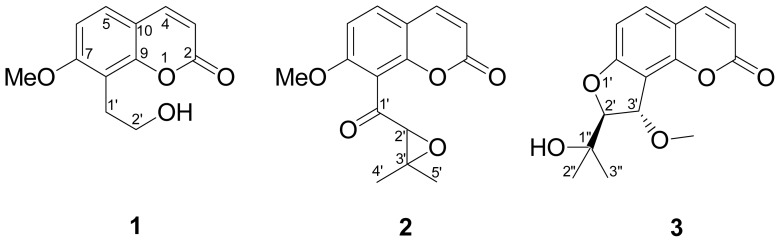
The chemical structures of new compounds **1**–**3** isolated from *Cnidium monnieri*.

*Cnidium monnieri* (L.) Cusson, belonging to the Umbelliferae family, is native to China and is an important traditional Chinese medicinal plant. It is widely distributed in China and is also found in Korea, Mongolia, and Russia. The dried fruits of *C. monnieri*, known in Chinese as “Shechuangzi”, have been used as traditional remedies for the skin disease, gynecopathy, and stasis of the blood [1]. Several investigations have reported that the fruits of *C. monnieri* exhibited various pharmacological effects including antidermatophytic effect [2], antipruritic action [3], anti-allergic effect [4], antiosteoporosis [5], antiproliferation of vascular smooth muscle cells [6], vasorelaxation [7], antifibrotic activity in hepatic cells [8], and anti-adipogenic activity in 3T3-L1 cells [9]. In addition, the chemical constituents including coumarins [10], chromones [11], and sesquiterpens [12], have been isolated from the fruits of *C. monnieri*. The above-mentioned beneficial effects are suggested to be due to coumarin compounds existing in the dried fruits of *C. monnieri* [6] and more than twenty compounds, such as osthole, edultin bergapten, isopimpineline, cnidiadin, archangelicin, imperatorin, xanthotoxin, oroselone, colnmbianadin, *O*-acetylcolnmbianetin and 2′-acetylangelicin were found. The 8-alkylcoumarin compound, osthole, has been proven to regulate cardiac or hepatic oxidative stress by Zhou and Zhang [13,14]. The oxidative stress is considered to be one of the most important factors for neurodegenerative disease like Alzheimer’s disease [15], thus in this study we aimed to explore the potential antioxidant candidates from the fruits of *C. monnieri*. Herein, we report the extraction, purification, structural elucidation, and cytoprotective activity of three new 8-alkylcoumarins (**1**–**3**) (Figure 1).

## 2. Results and Discussion

### 2.1. Isolation and Structural Elucidation

The MeOH extract of fruits of *C. monnieri* was concentrated to give a brown residue which was suspended in water and parititioned with EtOAc and *n*-BuOH, successively. The combined EtOAc soluble layer was subjected to repeated chromatography using silica gel and further purification by semipreparative HPLC (high-performance liquid chromatography) to furnish three new compounds, 7-*O*-methylphellodenol-B (**1**), 7-methoxy-8-(3-methyl-2,3-epoxy-1-oxobutyl)chromen-2-one (**2**), and 3′-*O*-methylvaginol (**3**) (Figure 1), in addition to seven known compounds, 7-methoxy-8-formylcoumarin (**4**) [16], hassanon (**5**) [17], *E*-murraol (**6**) [18], *Z*-murraol (**7**) [19], micromarin-F (**8**) [20], meranziz hydrate (**9**) [21], and albiflorin-3 (**10**) [22]. The identification of the known compounds were performed by comparing their physical and spectral data (IR (infrared), UV (ultraviolet), MS (mass spectrum), and NMR (nuclear magnetic resonance)) with literature values.

Compound **1** was obtained as a light yellow solid, and the high resolution electron impact mass spectrometry (HR-EI-MS) data determined the molecular formula to be C_12_H_12_O_4_ (*m*/*z* 220.0732 ([M]^+^, calcd 220.0736)), indicating seven degrees of unsaturation. The IR spectrum indicated the presence of hydroxyl (3250 cm^−1^), carbonyl (1719 cm^−1^), and aromatic (3065, 1606 and 1500 cm^−1^) functionalities.

**Figure 2.**
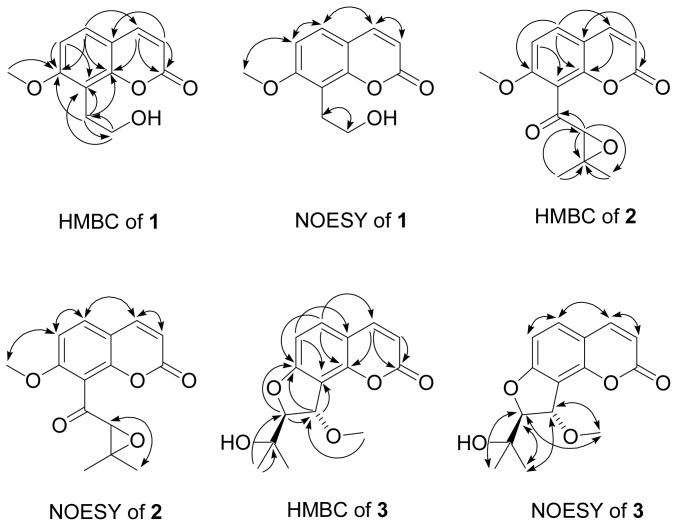
Significant heteronuclear multiple bond coherence (HMBC) and nuclear Overhauser enhancement spectroscopy (NOESY) correlations (two-headed arrows) of compounds **1**–**3**.

The ^1^H- and ^13^C-NMR spectra of **1** (Table 1) displayed signals characteristic of the presence of a methoxy [δ_H_ 3.91 (3H, s); δ_C_ 56.1 (q)], a (*Z*)-configured conjugated double bond of a six-membered ring [δ_H_ 6.23 (1H, d, *J* = 9.5 Hz, H-3), 7.61 (1H, d, *J* = 9.5 Hz, H-4); δ_C_ 113.1 (d), 143.8 (d)], two *ortho*-coupled aromatic protons [δ_H_ 7.32 (1H, d, *J* = 8.6 Hz, H-5), 6.84 (1H, d, *J* = 8.6 Hz, H-6); δ_C_ 127.0 (d), 107.3 (d)], and a hydroxyethyl group attached on the benzene ring [δ_H_ 3.15 (2H, t, *J* = 6.7 Hz, H-1′), 3.84 (1H, d, *J* = 6.7 Hz, H-2′); δ_C_ 26.3 (t), 62.0 (t)]. Twelve carbon signals were found in the ^13^C-NMR spectrum of **1** and were assigned by the distortionless enhancement by polarization transfer (DEPT) experiments as one aliphatic methylene, four olefinic methine, four quaternary olefinic, one secondary oxygenated, one carbonyl, and one methoxy carbons. On the basis of above spectral evidences, compound **1** was tentatively proposed to be a coumarin derivative exhibiting a methoxyl group neighbouring a hydroxyethyl group bonded to the benzene ring. The heteronuclear multiple bond coherence (HMBC) correlations (Figure 2) between H-4 (δ_H_ 7.61)/C-2 (δ_C_ 161.3 (s)), C-5 (δ_C_ 127.0 (d)), and C-9 (δ_C_ 153.4 (d)); H-5 (δ_H_ 7.32)/C-7 (δ_C_ 160.7 (t)) and C-9; and H-6 (δ_H_ 6.84)/C-8 (δ_C_ 114.9 (s)) confirmed the coumarin skeletal structure of **1**. The HMBC correlations between 7-OMe (δ_H_ 3.91)/C-7; H-1′ (δ_H_ 3.15)/C-7, C-8, and C-9 indicated that the methoxyl group and hydroxyethyl group were attached on C-7 and C-8, respectively. The significant nuclear Overhauser enhancement spectroscopy (NOESY) correlations between H-4/H-5 and H-6/7-OMe further confirmed the above proposal structure (Figure 2). Therefore compound **1** was determined as 7-*O*-methylphellodenol-B. Complete ^1^H- and ^13^C-NMR chemical shifts were established by ^1^H–^1^H correlated spectroscopy (^1^H–^1^H COSY), heteronuclear multiple-quantum coherence (HMQC), HMBC, and NOESY spectra.

Compound **2** was obtained as a yellow oil, and the high resolution electron impact mass spectrometry (HR-EI-MS) data determined the molecular formula to be C_15_H_14_O_5_ (*m*/*z* 274.0834 ([M]^+^, calcd 274.0841)), indicating nine degrees of unsaturation. The IR spectrum indicated the presence of two carbonyl (1732 and 1716 cm^−1^), and aromatic (3061, 1600 and 1507 cm^−1^) functionalities. The ^1^H- and ^13^C-NMR spectra of **2** (Table 1) revealed resonances for a methoxy [δ_H_ 3.92 (3H, s); δ_C_ 56.6 (q)], a (*Z*)-configured conjugated double bond of a six-membered ring [δ_H_ 6.27 (1H, d, *J* = 9.6 Hz), 7.62 (1H, d, *J* = 9.6 Hz); δ_C_ 113.4 (d), 142.8 (d)], two *ortho*-coupled aromatic protons [δ_H_ 7.51 (1H, d, *J* = 8.8 Hz), 6.89 (1H, d, *J* = 8.8 Hz); δ_C_ 130.9 (d), 104.3 (d)], a conjugated ketone carbonyl [δ_C_ 195.2 (s)], an oxymethine [δ_H_ 3.83 (1H, s); δ_C_ 66.8 (d)], and a quaternary oxygenated carbon [δ_C_ 63.7 (s)] linked with two methyls [δ_H_ 1.45, 1.52 (each 3H, s); δ_C_ 18.5 (q), 24.8 (q)]. Altogether, 15 carbon signals were observed in the ^13^C-NMR spectrum of **2** and were assigned by DEPT (distortionless enhancement by polarization transfer) experiments as two alphatic methyl, four olefinic methine, four quaternary olefinic, one tertiary oxygenated, one quaternary oxygenated, two carbonyl, and one methoxy carbons. By comparison of the ^1^H- and ^13^C-NMR data with those of **1**, indicated that both compounds exhibited identical structure in coumarin skeleton, the obvious differences occur in the signals of side chain at C-8. The NMR signals of hydroxyethyl group at C-8 were absent in **1**, replaced by that of a side chain composed of five carbons including a conjugated ketone carbonyl [δ_C_ 195.2 (s)], an oxymethine [δ_H_ 3.83 (1H, s); δ_C_ 66.8 (d)], and a quaternary oxygenated carbon [δ_C_ 63.7 (s)] linked with two methyls [δ_H_ 1.45, 1.52 (each 3H, s); δ_C_ 18.5 (q), 24.8 (q)]. The structure of side chain was determined as 3-methyl-2,3-epoxy-1-oxobutyryl moiety by HMBC correlations as follows: H-2′ (δ_H_ 3.83)/C-1′ (δ_C_ 195.2), C-3′ (δ_C_ 63.7), C-4′ (δ_C_ 18.5), and C-5′ (δ_C_ 24.8) and H-4′ (δ_H_ 1.45)/C-2′ (δ_C_ 66.8) and C-3′ (Figure 2). Thus, compound **2** was accordingly determined to be 7-methoxy-8-(3-methyl-2,3-epoxy-3-oxobutyl)chromen-2-one.

Compound **3** was obtained as a light yellow solid. The IR spectrum of **3** showed bands that were attributable to hydroxyl (3462 cm^−1^), carbonyl (1725 cm^−1^), and aromatic (3055, 1615, 1460 cm^−1^) functionalities. The ^1^H- and ^13^C-NMR spectra of **3** (Table 1) revealed resonances for a methoxy [δ_H_ 3.63 (3H, s); δ_C_ 57.9 (q)], a (*Z*)-configured conjugated double bond of a six-membered ring [δ_H_ 6.23 (1H, d, *J* = 9.4 Hz), 7.62 (1H, d, *J* = 9.4 Hz); δ_C_ 112.4 (d), 143.4 (d)], two *ortho*-coupled protons [δ_H_ 7.37 (1H, d, *J* = 8.4 Hz), 6.80 (1H, d, *J* = 8.4 Hz); δ_C_ 131.8 (d), 107.3 (d)], and two oxymethines [δ_H_ 4.51 (1H, d, *J* = 2.8 Hz), 5.21 (1H, d, *J* = 2.8 Hz); δ_C_ 96.1 (d), 79.4 (d)], and a quaternary oxygenated carbon [δ_C_ 71.4 (s)] linked to two methyls [δ_H_ 1.25, 1.31 (each 3H, s); δ_C_ 25.2 (q), 25.7 (q)]. The ^13^C-NMR spectrum displayed 15 resonances, which were differentiated by DEPT experiments into two aliphatic methyl, four olefinic methine, four quaternary olefinic, two tertiary oxygenated, one quaternary oxygenated, one carbonyl, and one methoxy carbons. These data suggested that **3** was a coumarin similar to compound **2**. The HR-EI-MS of **3** showed a molecular ion at *m*/*z* 276.0999, which corresponded to the molecular formula, C_15_H_16_O_5_, indicating eight degrees of unsaturation. Seven of eight degrees of unsaturation attributed to the basic structure of coumarin and the remaining one degree of unsaturation suggested two substituents of C-7 and C-8 should exhibit a ring structure. The two vicinal oxymethines [δ_H_ 4.51 (1H, d, *J* = 2.8 Hz), 5.21 (1H, d, *J* = 2.8 Hz); δ_C_ 96.1 (d), 79.4 (d)], a downshifted methoxy [δ_H_ 3.63 (3H, s); δ_C_ 57.9 (q)] attached on a aliphatic carbon, and two methyls [δ_H_ 1.45, 1.52 (each 3H, s); δ_C_ 18.5, 24.8 (q)] linked to an oxygenated quaternary carbon [δ_C_ 63.7 (s)] indicated that **3** exhibited a dihydrofuran structure bonded to C-7 and C-8 with two substituents, a methoxy and a 2-hydroxyisopropyl moiety. The proposal structure was confirmed by HMBC correlations as follows: H-2′ (δ_H_ 4.51)/C-7 and C-3′ (δ_C_ 79.4); H-3′ (δ_H_ 5.21)/C-7 (δ_C_ 163.9), C-8 (δ_C_ 112.9), and C-1″ (δ_C_ 71.4); H-2″ (δ_H_ 1.25)/C-2′ and C-1″; 3′-OMe (δ_H_ 3.63)/C-3′ (Figure 2). The *trans*-configuration at C-2′ and C-3′ was determined by significant NOE (nuclear Overhauser enhancement) correlations between H-2′/3′-OMe and H-3′/H-3″ in the NOESY spectrum. No NOESY correlation was found between H-2′ and H-3′ that further confirmed this assignment (Figure 2). Accordingly, compound **3** was determined to be 3′-*O*-methylvaginol.

### 2.2. Cytoprotective Activity against Oxidative Stress

**Figure 3.**
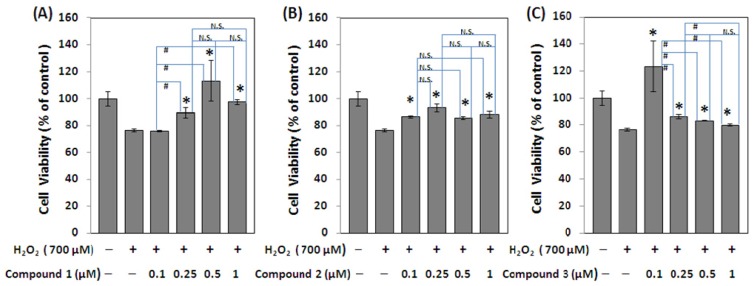
Protective effect from H_2_O_2_-induced oxidative stress on Neuro-2a neuroblastoma cells of (**A**) compound **1**; (**B**) compound **2**; and (**C**) compound **3**. The cell viability is expressed as a percentage relative to control without H_2_O_2_ treatment. Values are mean ± SEM from three experiments. The asterisk symbol (*) indicates a statistically significant difference (*p* < 0.05) in relation to control with H_2_O_2_ treatment. The symbol (#) indicates a statistically significant difference (*p* < 0.05) between each experimental group. N.S. means not significant.

The oxidative stress is considered to be one of the most important factors for neurodegenerative disease like Alzheimer’s disease [15]. H_2_O_2_-treatment has been shown to cause oxidative stress to the cells in culture [23] and impaired their proliferation. The cytoprotective activity against oxidative stress induced by hydrogen peroxide on Neuro-2a cells was assayed. All the isolates were evaluated the anti-oxidative activity by MTS (3-(4,5-dimethylthiazol-2-yl)-5-(3-carboxymethoxyphenyl)-2-(4-sulfophenyl)-2*H*-tetrazolium) cell proliferation assay and the results showed that all these three new 8-alkylcoumarins exhibited cytoprotective effect on Neuro-2a cells injured by hydrogen peroxide. The range of effective cytoprotective dosage for compound **1** was from 0.25 to 1 μM. Interestingly, compound **3** dramatically increased the cytoprotective effect at a lower dosage, 0.1 μM. Compound **2** showed slight cytoprotective ability at the concentration ranging from 0.1 to 1 μM (Figure 3). The remaining seven known compounds (**4**–**10**) exhibited no significant cytoprotective activity.

## 3. Experimental Section

### 3.1. Chemicals

Thirty percent hydrogen peroxide stock solution, 3-(4,5-dimethylthiazol-2-yl)-5-(3-carboxy methoxyphenyl)-2-(4-sulfophenyl)-2*H*-tetrazolium (MTS) and other chemicals were purchased from Sigma-Aldrich Chemical Co. (St. Louis, MO, USA).

### 3.2. General

UV spectra were obtained using a Shimadzu Pharmaspec-1700 UV-Visible spectrophotometer. Optical rotations were measured with a Jasco-DIP-180 polarimeter (JASCO, Inc., Tokyo, Japan). Infrared spectra were recorded on a Perkin-Elmer-983G FT-IR spectrophotometer (PerkinElmer Ltd., Bucks, UK). 1D- and 2D-NMR spectra were measured with a Varian-Unity-Plus-400 spectrometer or a Bruker DRX-500 FT-NMR spectrometer with tetramethylsilane (TMS) as the internal standard (Bruker Instruments, Karlsruhe, Germany). EI-MS and HR-EI-MS were measured with a JEOL SX-102A mass spectrometer and a Finnigan TSQ-46C mass spectrometer, respectively (Finnigan MAT, Inc., San Jose, CA, USA). Column chromatography was performed using Merck Si gel (230–400 mesh; Merck & Co., Inc., White House Station, NJ, USA), and TLC (thin-layer chromatography) analysis was carried out using aluminum pre-coated Si plates (Silica Gel 60 F-254; Merck & Co., Inc.); the spots were detected by spraying with 5% H_2_SO_4_ and then heating at 100 °C. Semi-preparative HPLC was performed using a normal phase column (LiChrosorb Si 60, 7 μm, 250 mm × 10 mm; Merck & Co., Inc.) on a LDC Analytical-III system.

### 3.3. Plant Material

The fruits of *C. monnieri* were purchased from a local medicine store in Taipei, Taiwan. The material was identified by Prof. Chao-Lin Kuo, Department of Chinese Pharmaceutical Science and Chinese Medicine Resources, China Medical University.

### 3.4. Extraction and Isolation

Dried fruits (6.45 kg) of *C. monnieri* (L.) were extracted with MeOH (40 L) two times (7 days each time) at room temperature. After removal of the solvent under vacuum, the extract was suspended in water (1 L), and then partitioned with EtOAc (1 L × 3) to yield a brown residue (338 g). The EtOAc fraction was chromatographed on silica gel (4.2 kg, 120 mm × 10 cm) using *n*-hexane–EtOAc and EtOAc–MeOH mixtures as solvent systems to obtain 11 fractions. Fraction 6 from *n*-hexane–EtOAc (6:4) elution was further chromatographed on a silica gel column (5 mm × 45 cm), eluted with CH_2_Cl_2_–EtOAc (8:1 to 0:1) to give seven fractions (each about 500 mL), 6A–6G. Fraction 6A was subjected to semipreparative HPLC eluted with *n*-hexane–EtOAc (7:3) and *n*-hexane–CH_2_Cl_2_–EtOAc (3:3:4) and to yield **4** (27.9 mg, 0.00433‰). Fraction 6C was subjected to semipreparative HPLC eluted with CH_2_Cl_2_–EtOAc (4:1) and *n*-hexane–CH_2_Cl_2_–MeOH (11:8:1) and to yield **6** (4.8 mg, 0.00074‰), **7** (28.5 mg, 0.00442‰), and **8** (43.1 mg, 0.00668‰). Fraction 6D was subjected to semipreparative HPLC eluted with *n*-hexane–CH_2_Cl_2_–EtOAc (3:8:9) and *n*-hexane–CH_2_Cl_2_–MeOH (7:10:1) to yield **3** (1.2 mg, 0.00019‰), **9** (2.2 mg, 0.00034‰), and **10** (5.0 mg, 0.00077‰). Fraction 6E was subjected to semipreparative HPLC eluted with *n*-hexane–EtOAc (9:11) and *n*-hexane–CH_2_Cl_2_–MeOH (8:11:1) to yield **1** (27.9 mg, 0.00433‰), **2** (1.0 mg, 0.00016‰), and **5** (2.1 mg, 0.00033‰).

7-*O*-Methylphellodenol-B (**1**). Light yellow solid; mp: 213–215 °C; EI-MS (70 eV) *m*/*z* (rel. int.%): 220 ([M]^+^, 33), 190 (99), 189 (100), 175 (15), 161 (13), 131 (85); HR-EI-MS *m*/*z*: 220.0732 [M]^+^ (calcd for C_12_H_12_O_4_, 220.0736); UV λ_max_ (MeOH): 320, 255, 247, 218 nm; IR (KBr) *ν*_max_: 3250, 3065, 1719, 1606, 1500, 1275, 1255, 1102, 1009, 844 cm^−1; 1^H-NMR and ^13^C-NMR (500/125 MHz, in CDCl_3_): see Table 1.

7-Methoxy-8-(3-methyl-2,3-epoxy-1-oxobutyryl)chromen-2-one (**2**). Yellow oil; [α]^25^_D_ = −171.0° (*c* = 0.03, MeOH); EI-MS (70 eV) *m*/*z* (rel. int.%): 274 ([M]^+^, 24), 259 (23), 203 (100); HR-EI-MS *m*/*z*: 274.0834 [M]^+^ (calcd for C_15_H_14_O_5_, 274.0841); UV λ_max_ (MeOH): 322, 303, 231, 211 nm; IR (KBr) *ν*_max_: 3061, 1732, 1716, 1600, 1507, 1295, 1255, 1096, 837 cm^−1; 1^H-NMR and ^13^C-NMR (400/100 MHz, in CDCl_3_): see Table 1.

3′-*O*-Methylvaginol (**3**). Light yellow solid; mp: 155–157 °C; [α]^25^_D_ ≈ 0° (*c* = 0.04, MeOH); EI-MS (70 eV) *m*/*z* (rel. int.%): 276 ([M]^+^, 37), 243 (68), 187 (100), 158 (19); HR-EI-MS *m*/*z*: 276.0999 [M]^+^ (calcd for C_15_H_16_O_5_, 276.0997); UV λ_max_ (MeOH): 324, 259, 249, 217 nm; IR (KBr) *ν*_max_: 3462, 3055, 1725, 1615, 1460, 1255, 1116, 837 cm^−1; 1^H-NMR and ^13^C-NMR (400/100 MHz, in CDCl_3_): see Table 1.

### 3.5. Cell Culture

Neuro-2a neuroblastoma cells (BCRC 60026) was purchased from the Bioresources Collection and Research Center (BCRC, Hsinchu, Taiwan) of the Food Industry Research and Development Institute (Hsinchu, Taiwan). Cells were cultured in plastic dishes containing Dulbecco’s Modified Eagle Medium (DMEM, Sigma, St. Louis, MO, USA) supplemented with 10% fetal bovine serum (FBS, Sigma) in a CO_2_ incubator (5% CO_2_ in air) at 37 °C and subcultured every 3 days at a dilution of 1:3 using 0.05% trypsin-0.02% EDTA in Ca^2+^- and Mg^2+^-free phosphate-buffered saline (DPBS).

### 3.6. Cell Viability Assay

The 3-(4,5-dimethylthiazol-2-yl)-5-(3-carboxymethoxyphenyl)-2-(4-sulfophenyl)-2*H*-tetrazolium (MTS) assay was performed to determine the anti-oxidative effects of three new 8-alkylcoumarins on Neuro-2a cell viability according to the manufacturer’s protocol. Cells (10^4^) were cultured in 96-well plate containing DMEM (Dulbecco’s modified eagle’s medium) supplemented with 10% FBS for 1 day to become nearly confluent. Then, cells were cultured with different concentrations of the three new compounds (**1**–**3**) and seven known compounds (**4**–**10**). After 1 h, cells were cultured in the presence of 700 μM hydrogen peroxide (Sigma-Aldrich) for further 7 h. After that, the cells were washed with DPBS and 120 μL MTS solution. After 2 h incubation at 37 °C, the absorbance at 490 nm was read using a microplate reader (Molecular Devices, Sunnyvale, CA, USA). All tests were performed in triplicate. Results are expressed as the percentage relative to control without H_2_O_2_ treatment.

### 3.7. Statistical Analysis

Multiple comparisons within experimental groups were made using one-way analysis of variance (ANOVA) to determine differences between experimental treatments and control with H_2_O_2_ treatment. A level of *p* < 0.05 was set for significance for all tests, and all values are expressed as mean ± SEM.

## 4. Conclusions

Ten coumarins were isolated from the fruits of *C. monnieri.* Among them, 7-*O*-methylphellodenol-B (**1**), 7-methoxy-8-(3-methyl-2,3-epoxy-1-oxobutyl)chromen-2-one (**2**), and 3′-*O*-methylvaginol (**3**) are new compounds. This investigation of secondary metabolites may contribute to better understanding on the chemical characteristics of *C. monnieri*.

The three new compounds **1**–**3** exhibited significant cytoprotective activity (Figure 3). Compounds **1** and **3** showed stronger cytoprotective activity than compound **2**. The dose range of cytoprotective effect for compound **1** was from 0.25 to 1 μM, and no significant difference was observed between the different concentrations, 0.25, 0.5, and 1 μM through the ANOVA analysis. Compound **3** showed better cytoprotective effect at the low dosage (0.1 μM), which may be due to its good scavenging ability against hydrogen peroxide. However, at the higher dosage, the cytoprotective effect of compound **3** was dramatically decreased, which may be due to its intrinsic cytotoxicity. Compound **2** showed a weaker cytoprotective effect compared to that of compounds **1** and **3** (Figure 3). It may be explained by the intrinsic cytotoxicity of compound **2** contributed by its reactive epoxide group [24,25]. In conclusion, we found that these three new 8-alkylcoumarins from the fruits of *C. monnieri*, 7-*O*-methylphellodenol-B (**1**), 7-methoxy-8-(3-methyl-2,3-epoxy-1-oxobutyl)chromen-2-one (**2**), and 3′-*O*-methylvaginol (**3**) could effectively protect Neuro-2a neuroblastoma cells from oxidative damage at the specific dose range.

## Figures and Tables

**Table 1.** NMR (nuclear magnetic resonance) data (CDCl_3_) of compounds **1**–**3**. δ in ppm, *J* in Hz.

Position	Compound 1		Compound 2		Compound 3	
			
	δ_H_^a^	δ_C_^b^	δ_H_^c^	δ_C_^d^	δ_H_^c^	δ_C_^d^
1						
2		161.3		159.8		159.9
3	6.23 (d, *J* = 9.5)	113.1	6.27 (d, *J* = 9.6)	113.4	6.23 (d, *J* = 9.4)	112.4
4	7.61 (d, *J* = 9.5)	143.8	7.62 (d, *J* = 9.6)	142.8	7.62 (d, *J* = 9.4)	143.4
5	7.32 (d, *J* = 8.6)	127.0	7.51 (d, *J* = 8.8)	130.9	7.37 (d, *J* = 8.4)	131.8
6	6.84 (d, *J* = 8.6)	107.3	6.89 (d, *J* = 8.8)	104.3	6.80 (d, *J* = 8.4)	107.3
7		160.7		159.2		163.9
8		114.9		116.2		112.9
9		153.4		152.4		151.7
10		113.0		112.7		113.9
1′	3.15 (t, *J* = 6.7)	26.3		195.2		
2′	3.84 (t, *J* = 6.7)	62.0	3.83 (s)	66.8	4.51 (d, *J* = 2.8)	96.1
3′				63.7	5.21 (d, *J* = 2.8)	79.4
4′			1.45 (s)	18.5		
5′			1.52 (s)	24.8		
1″						71.4
2″					1.25 (s)	25.2
3″					1.31 (s)	25.7
OCH_3_	3.91 (s)	56.1	3.92 (s)	56.6	3.63 (s)	57.9

Recorded at ^a^ 500 MHz (^1^H); ^b^ 125 MHz (^13^C); ^c^ 400 MHz (^1^H); and ^d^ 100 MHz (^13^C).
